# The Neural Basis of Herding Decisions in Enterprise Clustering: An Event-Related Potential Study

**DOI:** 10.3389/fnins.2019.01175

**Published:** 2019-10-30

**Authors:** Wuke Zhang, Danping Yang, Jia Jin, Liuting Diao, Qingguo Ma

**Affiliations:** ^1^Business School, Ningbo University, Ningbo, China; ^2^Academy of Neuroeconomics and Neuromanagement, Ningbo University, Ningbo, China; ^3^School of Management, Zhejiang University, Hangzhou, China

**Keywords:** herding decisions, business behavior, extracted event-related potentials, N2, late positive potential

## Abstract

Herding behavior refers to the social phenomenon in which people are intensely influenced by the decisions and behaviors of others in the same group. Although several recent studies have explored the neural basis of herding decisions in people’s daily lives (e.g., consumption decisions), the neural processing of herding decisions underlying enterprise behavior is still unclear. To address this issue, this study extracted event-related potentials (ERPs) from electroencephalographic data when participants (i.e., top executives in real enterprises) performed a choice task in which they judged whether to let their enterprises settle in an industrial zone when the occupancy rate of the industrial zone was either low or high. The behavioral results showed that participants had a higher acceptance rate in the high occupancy rate condition than in the low one, suggesting the existence of herding tendency in top executives’ business decisions. The ERP results indicated that anticonformity choices induced a larger N2 amplitude than herding choices, demonstrating that participants might experience larger perceived risk and more decision conflict when they processed anticonformity choices. In contrast, we observed that herding choices induced a larger LPP amplitude than anticonformity choices, hinting that participants might experience better evaluation categorization and higher decision confidence when they processed herding choices. Based on these results, this study provides new insights into the neural basis of herding decisions made by top executives in business.

## Introduction

Herding behavior initially referred to the movements of a group of animals such as cattle or sheep (Xiong et al., 2016). Currently, this concept is also used to describe particular social phenomena, in which people are strongly influenced by others’ decisions and follow behaviors of others in the same group (Asch, 1956; Banerjee, 1992). In fact, herding effect widely exists in human society and has been found in consumer behavior (Chen et al., 2010; Moraes, 2016; Gao et al., 2017), stock markets (Chiang and Zheng, 2010; Blake et al., 2017; Kabir and Shakur, 2018), housing markets (Ngene et al., 2017), and group incidents (Xiong et al., 2016). In past decades, a large number of studies have explored the determinants of herding effect and its impact on individual decisions and behaviors using surveys and behavioral tests (Banerjee, 1992; Cipriani and Guarino, 2005; Chen, 2008; Pierdzioch and Stadtmann, 2010; Huang et al., 2015; Chong et al., 2017).

With the rapid development of interdisciplinary research, a few scholars have recently attempted to explore the underlying neural basis of herding decisions related to consumer behaviors and group incidents with the tools of cognitive neuroscience (Chen et al., 2010; Xiong et al., 2016; Shen et al., 2018). For instance, one study used event-related potentials (ERPs) to investigate the neural and psychological bases of consumers’ herding decisions in purchasing books online (Chen et al., 2010). The authors found that the higher the consistency was within the customer reviews, the more herding decisions were made and the higher the amplitude of late positive potential (LPP), which is a component of the ERP that is sensitive to categorization processes (Chen et al., 2010). A more recent study investigated the influence of emotion on herding decisions in group incidents with the use of ERPs (Xiong et al., 2016). The authors found that the early negativity (N2) was attenuated by an angry or sorrowful mood compared with a positive one. Furthermore, the reduction in the early negativity (N2) made people struggle less when making herding decisions in group incidents (Xiong et al., 2016).

Previous interdisciplinary studies on herding effect have mainly focused on people’s daily decisions, but have ignored potential herding decisions in enterprise behavior. Although enterprise decisions are influenced by various factors (e.g., market circumstances, behavior of competitors, and consumer preferences), these decisions are finally made by top executives based on related information (Orth, 2002). Due to the innate limitation of human’s ability in processing information (Simon, 1956), sometimes top executives may also make herding decisions (Lian, 2005). Thus, it is of great significance to further explore potential herding decisions of top executives and especially the associated underlying neural basis, which can contribute to a better understanding of the herding effect in enterprise behavior.

Industrial zones are one of the most representative types of enterprise cluster, and entering into an industrial zone can be seen as a typical kind of enterprise clustering (Peters et al., 2015). Considering that there are likely to be potential herding decisions in enterprise clustering (Lian, 2005), the current study uses decisions about whether to enter an industrial zone as the context to investigate the underlying neural and psychological basis of top executives’ herding decisions. Additionally, as the occupancy rate of an industrial zone is a general indicator of other enterprises’ clustering behaviors and can be easily simulated in the laboratory, this factor is chosen as the cue to evoke top executives’ herding decisions in the current study.

## Literature Review and Hypotheses Development

### Behavioral Hypothesis

People tend to believe what most others believe, even though these beliefs may not be true (Deutsch and Gerard, 1955; Chen et al., 2010; Shen et al., 2018). For instance, when the download counts of software on a large commercial online system were artificially increased by repeated downloading, substantially more downloads were initiated for this software than for matched software whose downloads had not been artificially manipulated (Hanson and Putler, 1996). Additionally, another study manipulated the sales of the same product with a randomized between-subjects design and found that the higher the sales were, the greater the purchase intentions (Chen, 2008). Regarding the current study, the abovementioned herding tendency leads to a possible decision-making pattern when top executives are making decisions about whether to introduce their enterprises into an industrial zone: when a considerable number of similar enterprises have entered into a particular industrial zone (i.e., a high occupancy rate), top executives are likely to believe that entering that industrial zone is a good choice for their enterprises’ future performance and eventually decide to enter. When the occupancy rate is low, top executives are likely to believe that entering the industrial zone is not a good choice (e.g., a high risk) and eventually decide to quit. The following hypothesis is thus proposed:

H1: The acceptance rate of entering an industrial zone will be higher in the high occupancy rate condition than in the low occupancy rate condition.

### ERP Hypotheses

Based on the proposed decision-making pattern mentioned above, there is likely to be a process of evaluative categorization about consistency with other enterprises. Meanwhile, as different business choices are related to different levels of risk and different levels of consistency with other enterprises, there is also a process of risk perception and conflict perception. Thus, in investigating the neural basis of top executives’ herding decisions in enterprise clustering, the present study focuses on two ERP components that have been frequently investigated in decision studies and that are closely related to the processing of decision conflict and risk (N2) and evaluative categorization LPP.

#### N2

N2 is a frequently studied ERP component in decision studies and typically peaks around 250–350 ms after the onset of a stimulus (Folstein and Van Petten, 2008; Wang et al., 2016; Jin et al., 2017). A considerable number of studies have consistently suggested that N2 is a conflict-related component and its amplitude is positively correlated with decisional conflict (Folstein and Van Petten, 2008; Spapé et al., 2011; Larson et al., 2012; Jin et al., 2017). Recently, a few studies have begun to suggest that N2 can also reflect decisional risk robustly (Ma et al., 2015; Wang et al., 2016; Jin et al., 2017), as higher perceived risk during decision-making will cause greater decision difficulty, which will further lead to increased decisional conflict (Wang et al., 2016; Jin et al., 2017). For instance, lower product ratings and lower sales, which indicate larger perceived risk and greater decisional conflict, will result in an enhanced N2 amplitude (Wang et al., 2016).

As people tend to believe what most others believe (Chen et al., 2010; Xiong et al., 2016; Gao et al., 2017), people seem to experience less perceived risk with herding choices than anticonformity ones. Meanwhile, as people also tend to stay consistent with the majority (Xiong et al., 2016), herding choices seem to result in less decisional conflict than anticonformity ones. In regard to the current study, we thus speculate that high occupancy rates, rather than low ones, will cause less decision conflict and perceived risk during the decision about whether to enter an industrial zone.

Specifically, we hypothesize the following:

H2: The N2 amplitude in the high occupancy rate condition will be smaller than that in the low occupancy rate condition.

#### LPP

Late positive potential is a late positive-going component that mainly distributes over the posterior scalp (Herring et al., 2011). Studies on decisions have recently reported that LPP can reflect the cognitive processes related to categorization before final decisions are made (Chen et al., 2010; Wang et al., 2016; Jin et al., 2017). These studies argued that increased LPP amplitudes were related to the stimuli of better evaluation categorization (Chen et al., 2010; Wang et al., 2016; Jin et al., 2017). For instance, product descriptions implying lower risk and better future performance were found to elicit larger amplitudes of LPP (Wang et al., 2016; Jin et al., 2017).

As people tend to believe what most others believe (Deutsch and Gerard, 1955; Chen et al., 2010; Shen et al., 2018), people seem to evaluate the majority’s choice better than the minority’s choice. In the current study, an industrial zone with a high occupancy rate is the majority’s preference, thus should lead to people’s better evaluation than an industrial zone with a low occupancy rate. The following hypothesis is thus proposed:

H3: The LPP amplitude in the high occupancy rate condition will be larger than that in the low occupancy rate condition.

## Materials and Methods

### General Experimental Design

This study adopted a randomized within-subjects repeated measure design. For each decision presented in this experiment, participants (i.e., top executives from textile enterprises) were asked to decide whether to let their enterprises settle in an industrial zone according to a specific occupancy rate. In order to facilitate the participation of real top executives, the current study chose the design of one independent variable (i.e., occupancy rate) with two levels rather than multiple levels to lessen participators’ consumption of time and vigor in the experiment. Besides, some previous ERP studies also applied the design of one independent variable with two levels to study decision-making (Jin et al., 2017, 2018). Thus, the occupancy rates used in the experiment included two levels (high vs. low). The categorization of high and low industrial zone occupancy rates was based on a former interview with managers from textile enterprises in Ningbo (a city characterized by the textile industry) in China. In order to better simulate the scenario, detailed information (excluding the occupancy rate) about a real textile industrial zone in Ningbo was offered to participants before they started the ERP experiment.

### Participants

Twenty-one top executives (17 males; 4 females) from twelve textile enterprises in Ningbo were recruited to participate in the current study. All of them were right-handed healthy native Chinese speakers with normal or corrected-to-normal vision, and none of them had a history of neurological disorder or mental disease. Their ages ranged from 22 to 57 years old, with a mean age of 37.81 years. The participants signed written informed consent forms before the study and were paid 500 Chinese yuan (approximately $73.70) as remuneration. The reason of the much higher remuneration than traditional subject compensation is that the participants in this study had much higher income levels than volunteers in typical ERP studies (most volunteers in ERP studies are university students), and the rate of remuneration/volunteers’ income in the current study is basically consistent with that in other ERP studies. Data of four participants (3 males; 1 female) were discarded because of excessive artifacts in the EEG recording. The present study was approved by the Internal Review Board of the Academy of Neuroeconomics and Neuromanagement in Ningbo University.

### Materials

In order to determine the standard for high vs. low industrial zone occupancy rates, a group interview of managers from textile enterprises in Ningbo was conducted. The results showed that managers in textile enterprises considered occupancy rates around 50% to be a low level and deemed occupancy rates around 90% to be a high level. Thus, thirty numbers from 48.5 to 51.5% (0.1% interval, without 50%) and thirty numbers from 88.5 to 91.5% (0.1% interval, without 90%) were used as the low occupancy rates and high occupancy rates, respectively, in the formal experiment.

### Procedure

Participants sat comfortably in a sound-attenuated room, about 100 cm away from a computer-controlled monitor (1280 × 1024 pixels) with a refresh rate of 60 Hz. Then, detailed information (excluding the occupancy rate) of a real textile industrial zone in Ningbo was presented to them. After a detailed reading of this information, participants were informed that they would next browse various occupancy rates, imagining that they were making a decision about whether to enter the mentioned textile industrial zone for each occupancy rate. Meanwhile, participants were also instructed to make their decisions using a wireless keypad.

**FIGURE 1:**
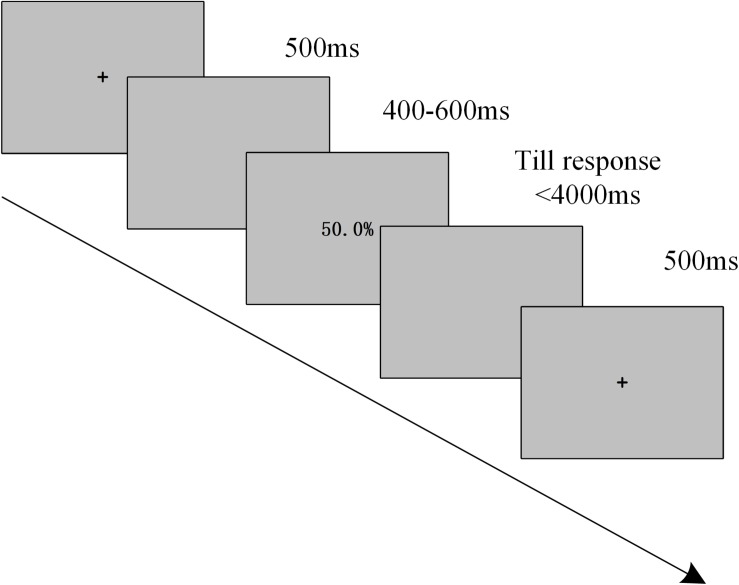
Experimental task: Participants were instructed to make decisions about whether to enter a textile industrial zone according to different occupancy rates.

On each trial in the formal experiment, a fixation (500 ms), a blank (400–600 ms), and a target stimulus of an occupancy rate (disappearing either after 4000 ms or when participants pressed the button) were presented sequentially (see Figure 1). The target stimuli included 30 low occupancy rates and 30 high occupancy rates, and each target stimulus of an occupancy rate appeared twice and was placed at the center of a gray background with 40 fonts, SimSun and font-weight. Thus, the formal experiment consisted of two blocks and each block included 60 trials. Additionally, all trials were presented randomly in the experiment.

Before the experiment, participants were instructed to press the button “1” if they decided to enter the industrial zone, or press the button “3” if they did not. E-Prime 2.0 software (Psychology Software tools, Pittsburgh, PA, United States) was used to present the stimuli and to record the triggers. Prior to the formal experiment, participants performed five trials to become familiarized with the task. The occupancy rates used in the practice trials were 10, 30, 50, 70, and 90%.

### Electroencephalogram (EEG) Recording and Analysis

A 64 Ag/AgCl electrodes cap was used to record electrooculograms with a Neuroscan Synamp2 Amplifier (Scan 4.3.1, Neurospft Labs, Inc). The forehead location and the left mastoid were used as the ground and the reference, respectively. Data was transferred to average off-line using left and right mastoid references. A pair of electrodes placed above and below the right eye were used to record the vertical EEG, and another pair of electrodes placed on the lateral canthi of both eyes was used to record the horizontal EEG. The experiment started when impedances of electrodes were below 5 kΩ.

Two hundred milliseconds prior to the onset of stimuli of occupancy rates to eight hundred milliseconds after this onset was used as the epoch, and 200 ms prior to the onset of stimuli was used as the baseline. The EEG was corrected using the method proposed by Semlitsch (Semlitsch et al., 1986). Trials were rejected when they had bursts of electromyography activity, peak-to-peak deflection exceeding ± 100 μV and amplifier clipping. Low pass filter at 30 Hz (24 dB/Octave) was used to digitally filter the average ERP.

According to previous research and the visual observation of the scalp distribution responding to the grand average waveforms in our study, nine electrodes (F1, Fz, F2, FC1, FCz, FC2, C1, Cz, and C2) were chosen for the N2 component analysis, and another nine electrodes (C1, Cz, C2, CP1, CPz, CP2, P1, Pz, and P2) were chosen for the LPP component analysis. In addition, the time window responded to the N2 and LPP components was 260–320 ms and 350–500 ms, respectively. Then, a 2 (occupancy rate: low level vs. high level) × 3 (frontal-to-central electrode: frontal vs. frontal-central vs. central) × 3 (left-middle-right electrode: left vs. middle vs. right) ANOVA (analysis of variance) was performed for the mean amplitude of the N2 component, while another 2 (occupancy rate: low level vs. high level) × 3 (central-to-parietal electrode: central vs. central-parietal vs. parietal) × 3 (left-middle-right electrode: left vs. middle vs. right) ANOVA was performed for the mean amplitude of the LPP component. Greenhouse-Geisser corrections were used for determining significances (Greenhouse and Geisser, 1959), and partial eta-squared values (η^2^_p_) are reported to demonstrate the effect sizes in ANOVA models (Cohen, 1988). In addition, Bonferroni method was performed for multiple comparisons in this study.

## Results

### Behavioral Results

A pairwise *t*-test was performed to analyze response times and acceptance rates. For response times, there was no significant difference between the high occupancy rate condition and the low occupancy rate condition [*M*_high_ = 706.359 ms, SE = 89.064; *M*_low_ = 753.191 ms, SE = 100.486; *t*(1,16) = 1.445, *p* = 0.168]. For acceptance rates, there was a significant difference: participants exhibited a higher acceptance rate in the high occupancy rate condition relative to the low occupancy rate condition [*M*_high_ = 87.7%, SE = 0.056; *M*_low_ = 62.8%, SE = 0.092; *t*(1,16) = 2.375, *p* = 0.030]. Thus, H1 is supported.

### ERP Results

#### N2

The results of a 2 (occupancy rate: low level vs. high level) × 3 (frontal-to-central electrode: frontal vs. frontal-central vs. central) × 3 (left-middle-right electrode: left vs. middle vs. right) ANOVA showed a significant main effect of occupancy rate [*F*(1,16) = 9.832, *p* = 0.006,η^2^_p_ = 0.381], suggesting that a larger mean amplitude of N2 was elicited in the low occupancy rate condition (i.e., anticonformity choices) (*M*_low_ = –0.152 mV, SE = 0.493) than in the high occupancy rate condition (i.e., herding choices) (*M*_high_ = 1.043 mV, SE = 0.729). Meanwhile, we also found that the occupancy rate interacted significantly with the frontal-to-central electrode [*F*(1.65,26.50) = 8.164, *p* = 0.003, η^2^_p_ = 0.338]. A follow-up simple effect analysis found that participants elicited a larger N2 amplitude for anticonformity choices than for herding choices in all frontal (*M*_low_ = −0.105, SE = 0.474; *M*_high_ = 1.048, SE = 0.687, *p* = 0.009), frontal-central (*M*_low_ = −0.175, SE = 0.538; *M*_high_ = 0.671, SE = 0.783, *p* = 0.050), and central electrodes (*M*_low_ = −0.175, SE = 0.538; *M*_high_ = 1.411, SE = 0.772; *p* = 0.001), suggesting a similar pattern of neural activity between herding choices and anticonformity choices over the human frontal-to-central brain regions.

However, we didn’t find a significant main effect of the frontal-to-central electrode [*F*(2,32) = 0.951, *p* = 0.355, η^2^_p_ = 0.056] or the left-middle-right electrode [*F*(2,32) = 0.846, *p* = 0.404, η^2^_p_ = 0.050]. In addition, the interaction effect between occupancy rate and the left-middle-right electrode was also not significant [*F*(2,32) = 0.652, *p* = 0.475, η^2^_p_ = 0.039], as well as the interaction effect between occupancy rate, the frontal-to-central electrode and the left-middle-right electrode [*F*(4,64) = 2.505, *p* = 0.080, η^2^_p_ = 0.135].

**FIGURE 2:**
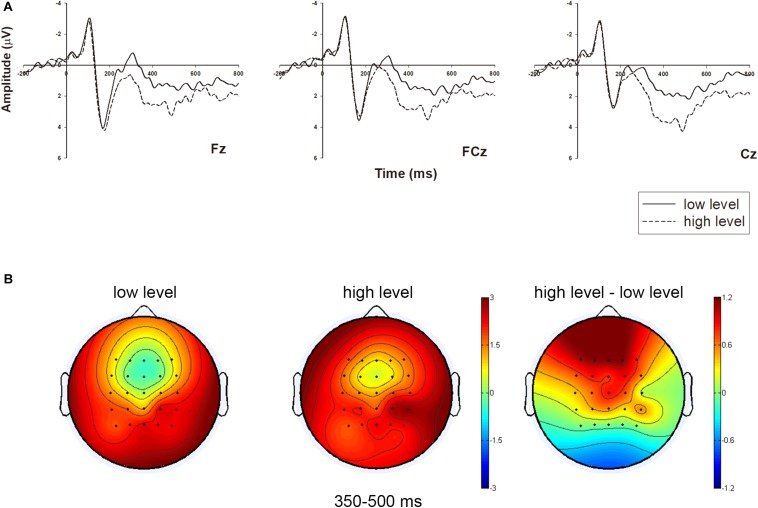
Grand-averaged event-related potential (ERP) waveforms of N2 in the frontal-to-central region with three electrodes, and related brain topography. **(A)** The N2 amplitude comparison of the two occupancy rate conditions (low level vs. high level) in representative electrodes (Fz, FCz, and Cz). **(B)** The brain topography of the two conditions and contrast at the N2 time window of 260–320 ms.

These results of N2 support H2. We chose three middle-line electrodes (i.e., Fz, FCz, and Cz) and illustrated their neural dynamic activity under different occupancy rate conditions in Figure 2A. Meanwhile, the brain topography was shown in Figure 2B, which showed the main difference between the two conditions in the frontal-to-central region.

#### LPP

A 2 (occupancy rate: low level vs. high level) × 3 (central-to-parietal electrode: central vs. central-parietal vs. parietal) × 3 (left-middle-right electrode: left vs. middle vs. right) ANOVA showed a significant main effect of occupancy rate [*F*(1,16) = 7.680, *p* = 0.014, η^2^_p_ = 0.324], indicating that a larger LPP amplitude was elicited in the high occupancy rate condition (i.e., herding choices) (*M*_high_ = 3.727 μV, SE = 0.874) than in the low occupancy rate condition (i.e., anticonformity choices) (*M*_low_ = 2.306 μV, SE = 0.599). Moreover, we found that the occupancy rate interacted significantly with the central-to-parietal electrode [*F*(1.445,23.116) = 8.566, *p* = 0.004, η^2^_p_ = 0.349]. A follow-up simple effect analysis found that participant elicited a larger LPP amplitude for herding choices than for anticonformity choices in central (*M*_high_ = 3.693, SE = 1.084; *M*_low_ = 1.923, SE = 0.738; *p* = 0.007), central-parietal (*M*_high_ = 4.113, SE = 0.966; *M*_low_ = 2.567, SE = 0.639; *p* = 0.013) and parietal electrodes (*M*_high_ = 3.375, SE = 0.766; *M*_low_ = 2.428, SE = 0.602; *p* = 0.048), suggesting a similar pattern of neural activity between herding choices and anticonformity choices over the human central-parietal brain regions.

However, we didn’t find a significant main effect of the central-to-parietal electrode [*F*(2,32) = 0.520, *p* = 0.489, η^2^_p_ = 0.031], or the left-middle-right electrode [*F*(2,32) = 1.006, *p* = 0.352, η^2^_p_ = 0.059]. In addition, the interaction effect between occupancy rate and the left-middle-right electrode was also not significant [*F*(2,32) = 0.570, *p* = 0.537, η^2^_p_ = 0.034], as well as the interaction effect among occupancy rate, the central-to-parietal electrode and the left-middle-right electrode [*F*(4,64) = 0.654, *p* = 0.532, η^2^_p_ = 0.039].

**FIGURE 3:**
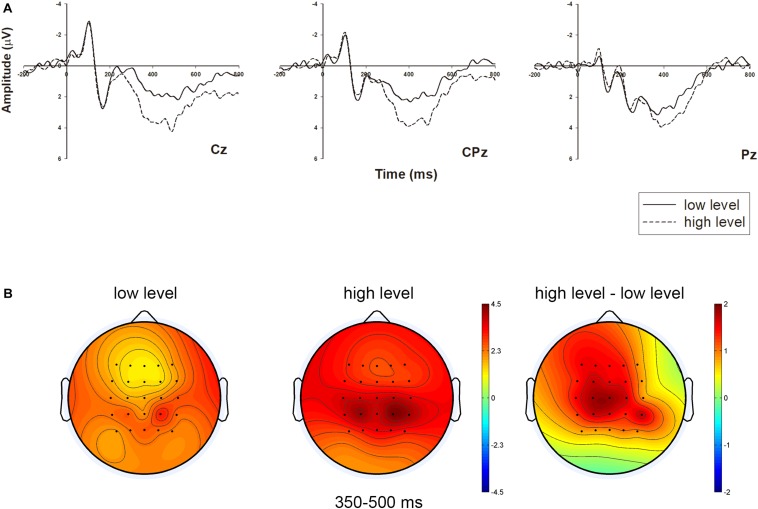
Grand-averaged ERP waveforms of LPP in the central-to-parietal region with three electrodes, and related brain topography. **(A)** The LPP amplitude comparison of the two occupancy rate conditions (low level vs. high level) in representative electrodes (Cz, CPz, and Pz). **(B)** The brain topography of the two conditions and contrast at the LPP time window of 350–500 ms.

These results of LPP support H3. We chose three middle-line electrodes (i.e., Cz, CPz, and Pz) and illustrated their neural dynamic activity under different occupancy rate conditions in Figure 3A. Meanwhile, the brain topography was shown in Figure 3B, which showed the main difference between the two conditions in the central-to-parietal region.

#### Correlation Analysis of N2 and LPP

**FIGURE 4:**
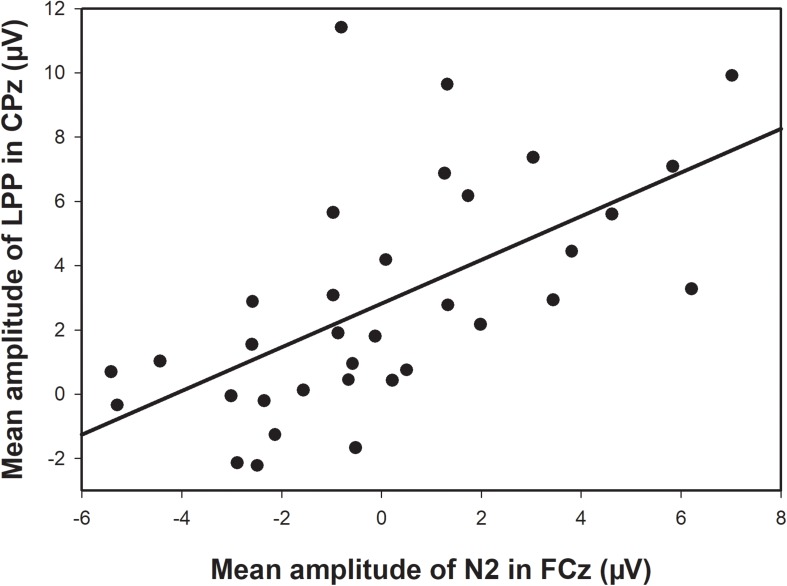
The correlation between amplitudes of N2 and LPP: Linear correlation between the mean amplitude of N2 in the FCz electrode and the mean amplitude of LPP in the CPz electrode.

A correlation analysis between the mean amplitudes of N2 and LPP was conducted. The results showed that there was a significant positive correlation (*r* = 0.654, *p* < 0.001) between the mean amplitude of N2 in the FCz electrode and the mean amplitude of LPP in the CPz electrode (see Figure 4).

## Discussion

The present study attempted to investigate the neural basis of herding decisions in enterprise clustering by detecting top executives’ decisions about whether to enter an industrial zone. Meanwhile, the industrial zone’s occupancy rate was chosen as the cue to evoke top executives’ herding decisions.

The behavioral results showed that participants had a higher acceptance rate in the high occupancy rate condition than in the low occupancy rate condition, demonstrating that the occupancy rate is likely to be an appropriate cue to evoke herding decisions in the context of the current study. More importantly, the behavioral results preliminarily proved the existence of herding tendency in top executives’ business decisions. In modern enterprises, top executives tend to make decisions based on various types of business information (Orth, 2002). However, due to the infinity of business information and the innate limitations of human’s ability in processing information (Simon, 1956), top executives cannot obtain or process all related information when making business decisions. Therefore, in some situations, they tend to believe what most others believe and do what most others do. In regard to the present study, although top executives were offered detailed information about a real textile industrial zone in Ningbo, they still might not be sure whether it was a suitable choice to enter the industrial zone. After all, some latent information about this industrial zone and the surrounding business environment was not fully obtained. Thus, top executives were likely to make decisions (partly) based on other enterprises’ behaviors: if only a small number of enterprises had entered the industrial zone, they might believe that entering the industrial zone would be a risky choice and would not be good for their enterprises’ future performance; in the end, they decided to decline these ventures. Inversely, if a considerable number of related enterprises had entered, the executives were likely to believe that entering the industrial zone was a good choice for their enterprises’ future performance and eventually decided to enter.

The event-related potentials results showed that herding choices elicited a smaller N2 amplitude and a larger LPP amplitude than the anticonformity choices. These results can be explained as follows. First, people tend to believe what most others believe (Chen et al., 2010; Xiong et al., 2016; Gao et al., 2017). Participants also tended to believe that entering an industrial zone with a high occupancy rate was not risky and would be helpful for their enterprises’ future performance. Inversely, participants tended to believe that it was a risky choice to enter an industrial zone with a low occupancy rate. As higher perceived risk during decision-making will cause increased decision difficulty and will further lead to increased decisional conflict (Wang et al., 2016; Jin et al., 2017), a larger N2 amplitude was evoked in the low occupancy rate condition (i.e., anticonformity choices) rather than in the high occupancy rate condition (i.e., herding choices). Second, people tend to remain consistent with most others (Chen et al., 2010; Xiong et al., 2016; Gao et al., 2017). If a choice is inconsistent with most others’ behavior, response conflict will be enhanced (Xiong et al., 2016). As response conflict can positively influence the amplitude of N2 (Gajewski et al., 2016; Jin et al., 2017), a larger N2 amplitude may be induced by anticonformity choices than by herding choices. Together, anticonformity choices (compared to herding choices) led to higher perceived risk and higher response conflict, both of which were reflected by a larger N2 amplitude.

We also observed that herding choices elicited a larger LPP amplitude than anticonformity choices. A considerable number of studies have found that LPP component may reflect the neurophysiological mechanism of categorical processing (Chen et al., 2006; Schupp et al., 2014; daSilva et al., 2016; Delaney-Busch et al., 2016; Pollux, 2016), and the stimuli leading to better evaluation categorization will elicit an increased LPP amplitude (Chen et al., 2010; Wang et al., 2016; Jin et al., 2017). As people tend to believe what most others believe (Deutsch and Gerard, 1955; Chen et al., 2010; Shen et al., 2018), participants in this study were likely to evaluate an industrial zone with a high occupancy rate better and classify it as a categorization of better evaluation (compared with an industrial zone with a low occupancy rate), which was mirrored by a larger LPP amplitude. In addition, several lines of evidence have suggested that the LPP amplitude in a decision task is positively related to decision confidence (Finnigan et al., 2002; Chen et al., 2010). As mentioned previously, participants’ perceived level of risk should be higher in the low occupancy rate condition than that in the high occupancy rate condition. In other words, decision confidence in the high occupancy rate condition should be higher than that in the low occupancy rate condition, as reflected by a larger LPP amplitude. Together, compared to the low occupancy level, the high occupancy level led to participants’ better evaluation categorization and higher decision confidence, both of which were reflected in a larger LPP amplitude.

The findings of the present study have some implications. The first is that herding effect should be further explored in other aspects of human society. Previous studies have found herding effect in consumer behaviors (Chen et al., 2010; Moraes, 2016; Gao et al., 2017), stock markets (Chiang and Zheng, 2010; Blake et al., 2017; Kabir and Shakur, 2018), housing markets (Ngene et al., 2017), and group incidents (Xiong et al., 2016). The present study preliminarily indicated the existence of herding effect in business decisions. Next, it is important to ask whether herding effect will exist in other aspects of human society, such as educational behavior (e.g., the selection of educational methods and tools) and patient behavior (e.g., the selection of hospitals and treatment programs). All of these questions require further exploration in future studies. The second implication concerns the application of neurocognitive tools in decision studies. Decision-making is a complex process, usually accompanied by implicit, and unconscious processes (Camerer and Yoon, 2015). In many situations, people are unable to articulate the reasons for their decisions and behaviors (Mueller et al., 2010; Camerer and Yoon, 2015). Neurocognitive tools, which can assess people’s neural bases during decision-making and deliver cognitive information (Chen et al., 2010; Mueller et al., 2010; Camerer and Yoon, 2015; Shen et al., 2018), will help scholars better understand people’s processes of decision-making and reveal the underlying neural and psychological mechanisms. Thus, if possible, researchers can combine neurocognitive tools (e.g., ERPs and functional magnetic resonance imaging) and traditional methods (e.g., survey and behavioral experiment) to conduct decision studies.

There are some limitations that have to be acknowledged. First, due to limited research resources, there was no control group in which the participants were gender-, education- and age-matched volunteers who were not top executives. This leads to an open question as to whether the findings are specific to top executives of enterprises or whether they could be generalized to a larger population. Second, there is no control condition in which the decisions are not associated with enterprises. Therefore, whether the herding choices of top executives are specifically due to their business expertise is still unclear. Third, although the present study recruited top executives from real enterprises as participants and simulated a real decision environment to the greatest extent, there was still a gap between the experimental scene and real decision circumstances. On the whole, one should be careful when generalizing the conclusions form this work to a larger population or to real world decisions.

## Conclusion

In summary, the current study investigated the neural basis of herding decisions in enterprise clustering. The behavioral results preliminarily proved the existence of herding tendency in top executives’ business decisions: participants exhibited a higher acceptance rate in the high occupancy rate condition than in the low occupancy rate condition. The ERP results indicated that anticonformity choices led to higher perceived risk and higher response conflict (reflected by a larger N2 amplitude) than herding choices. In contrast, herding choices led to participants’ better evaluation categorization and greater decision confidence (reflected by a larger LPP amplitude) compared to anticonformity choices.

## Data Availability Statement

The datasets generated for this study are available on request to the corresponding author.

## Ethics Statement

This study was carried out in accordance with the recommendations of the Ethics Committee of the Academy of Neuroeconomics and Neuromanagement at Ningbo University with written informed consent from all subjects. All subjects gave written informed consent in accordance with the Declaration of Helsinki. The protocol was approved by the Ethics Committee of the Academy of Neuroeconomics and Neuromanagement at Ningbo University.

## Author Contributions

WZ made substantial contributions to the work and participated in all aspects of the manuscript, conducted the experiment, analyzed the data, and wrote the manuscript. DY and JJ made substantial contributions to the work and participated in all aspects of the manuscript. LD participated in the data acquisition and data interpretation stage. QM oversaw the study and managed every part of the research. All authors read and approved the final manuscript.

## Conflict of Interest

The authors declare that the research was conducted in the absence of any commercial or financial relationships that could be construed as a potential conflict of interest.
